# Supplementary material for: Formulation and Development of Bioadhesive Oral Films Containing Usnea barbata (L.) F.H.Wigg Dry Ethanol Extract (F-UBE-HPC) with Antimicrobial and Anticancer Properties for Potential Use in Oral Cancer Complementary Therapy
Source: Pharmaceutics. 2022 Aug 28;14(9):1808. doi: 10.3390/pharmaceutics14091808 (PMC9505056; doi:10.3390/pharmaceutics14091808)
Supplement: Supplementary file 1 [file pharmaceutics-14-01808-s001.zip › Supplementary Material, Figure S1.pdf]

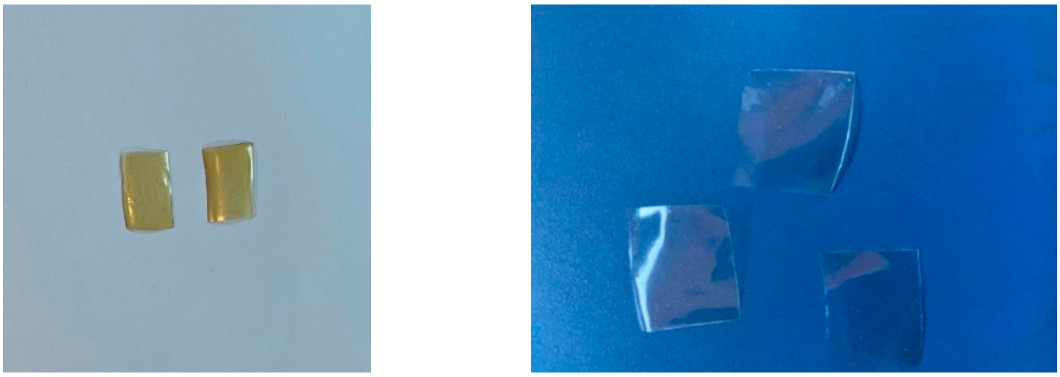

(**a**) (**b**)

Figure S1. Bioadhesive oral films F-UBE-HPC (a) and Reference (b); R—bioadhesive oral films without active lichen extract; F-UBE-HPC—bioadhesive oral film loaded with *U. barbata* dry ethanol extract.
